# CD30^+^OX40^+^ Treg is associated with improved overall survival in colorectal cancer

**DOI:** 10.1007/s00262-021-02859-x

**Published:** 2021-02-02

**Authors:** Jian Hang Lam, Michelle Hong, Si-Lin Koo, Clarinda Wei Ling Chua, Kah Ling Lim, Felicia Wee, Wei Keat Wan, Wei Qiang Leow, Joo Guan Yeo, Iain Bee Huat Tan, Joe Yeong, Tony Kiat Hon Lim, Tong Seng Lim

**Affiliations:** 1 A. Menarini Biomarkers Singapore Pte Ltd, Singapore, Singapore; 2 Division of Medical Oncology, National Cancer Centre, Singapore, Singapore; 3 Division of Pathology, Singapore General Hospital, Singapore, Singapore; 4 Translational Immunology Institute, SingHealth Duke-NUS Academic Medical Centre, Singapore, Singapore; 5 Institute of Molecular Cell Biology (IMCB), Agency of Science, Technology and Research (A*STAR), Singapore, Singapore

**Keywords:** Treg, Colorectal cancer, CD30, OX40, Diagnosis, Prognosis

## Abstract

Regulatory T cells (Tregs) are often enriched in tumors, where their immunosuppressive function has a key role in tumor persistence and progression. In colorectal cancer (CRC), however, Tregs are frequently associated with an improved clinical outcome. Tumor-infiltrating Tregs have been shown to exhibit a distinct signature comprising the co-stimulatory molecules (OX40, 4-1BB), cytokine receptors (IL1R2, IL21R, CCR8, CD30), and co-inhibitory molecules (PD-L1, TIGIT). Here, we showed by flow cytometry that circulating CD45RO^+^ Tregs from patients with CRC (*n* = 25) have elevated CD30 and OX40 expression compared to healthy subjects (*n* = 14). We identified co-expression of CD30 and OX40 on circulating CD45RO^+^ Tregs using single-cell images captured by the DEPArray^™^ system. The frequency of CD30^+^OX40^+^CD45RO^+^ Tregs was significantly higher in CRC patients than in healthy subjects (*P* < 0.001). Importantly, receiver operating characteristic analysis confirmed that this CD30^+^OX40^+^ Treg subset could strongly discriminate between CRC patients and healthy subjects with the highest accuracy of 92.3%, an AUC of 0.92, a sensitivity of 88%, a specificity of 100%, a positive predictive value of 100%, a negative predictive value of 82.35%, and a trade-off value of 3.44%, compared to other Treg subsets. Consistently, multiplex-IHC/IF of tumor-infiltrating Tregs revealed a significant association between high densities of CD30^+^OX40^+^ Tregs and improved overall survival; no such association was found for other subsets. These data suggest a potential role for CD30^+^OX40^+^ Tregs as a diagnostic or prognostic biomarker in CRC.

**Supplementary Information:**

The online version contains supplementary material available at 10.1007/s00262-021-02859-x.

## Introduction

Colorectal cancer (CRC) is a major health and socioeconomic problem worldwide. In Singapore, the incidence of CRC ranks among the highest in the world: 9320 new cases of CRC were diagnosed between 2010 and 2014, giving a crude incidence of 48.9 per 100,000 [1]. Early detection remains critical since prognosis is mostly dependent on the disease stage. Current screening methods include invasive imaging techniques (i.e., colonoscopy and sigmoidoscopy) and non-invasive stool-based tests [1, 2]. A blood-based liquid biopsy for CRC is desirable; however, few are commercially available. One notable example is the FDA-approved Epi proColon^®^, which is based on the detection of cell-free methylated SEPT9 DNA to diagnose CRC [3]. Another example is the FDA-approved CellSearch system that detects circulating tumor cells (CTCs), the frequency of which is correlated with disease stage and hence useful for prognostication [4].

CRCs, like many other human cancers, are enriched with tumor-infiltrating regulatory T cells (Tregs). Tregs are a distinct lineage of CD4^+^ T cells that possess potent immunosuppressive function. They are critical for maintaining immune homeostasis through suppression of auto-reactive immune cells and inhibition of the immune response at the resolution of infection [5]. These immunosuppressive functions of Tregs play a key role in tumor persistence and progression. Indeed, increased Treg infiltration in many cancers, including gastrointestinal, lung, ovarian, breast, pancreatic cancers, and melanoma [6–8], is generally associated with poor patient survival [9–11]. Conversely, many studies have established tumor-infiltrating Foxp3^+^ Tregs as a prognostic indicator of a favorable clinical outcome in CRC patients [12–15]. The reason for this seemingly paradoxical phenomenon remains unclear, but one possibility relates to the heterogeneity of Tregs.

Foxp3^+^ Tregs are not a uniform population but comprise subpopulations displaying distinct phenotypes and functions that may influence cancer progression differently [16–20]. Transcriptomic analysis of tumor-infiltrating Tregs in CRC patients of Caucasian origin has been performed by De Simone et al. [21] and validated by flow cytometry (FCM) at the single-cell level. Results from this study suggested that patients with CRC may harbor tumor-infiltrating Tregs with a specific protein signature, including the co-stimulatory molecules (OX40, 4-1BB), cytokine receptors (IL1R2, IL21R, CCR8, CD30), and co-inhibitory molecules (PD-L1, TIGIT). However, it is unknown whether this Treg signature can be seen in the peripheral blood of CRC patients.

In this study, we asked if circulating Tregs from CRC patients also exhibit this distinct expression profile and could serve as a cancer biomarker with clinical significance. We first confirmed this Treg signature in tumor samples from a South-East Asian cohort of CRC patients, and then determined the different Treg subsets in the blood of the CRC patients using FCM and the DEPArray™ system. We performed receiver operating characteristic (ROC) analyses to highlight the ability of the identified Treg subpopulations to discriminate between CRC patients and healthy subjects. Lastly, we determined the predictive effects of the different Treg subpopulations in CRC tumors on patients’ clinical outcomes. These data could help contribute to a better understanding of the heterogeneity of Tregs and may pave the way for the identification of novel biomarkers in CRC diagnosis.

## Materials and methods

### Patients

Two cohorts of CRC patients were studied: (1) a prospective cohort of 30 patients for FCM analysis, and (2) a retrospective cohort of 217 patients for tissue microarray and multiplex-IHC/IF studies. A total of 217 archival formalin-fixed, paraffin-embedded (FFPE) CRC specimens from patients diagnosed between January 2006 and December 2014 at the Department of Anatomical Pathology, Division of Pathology, Singapore General Hospital, were analyzed. Patient demographics, tumor histology, and stage of cancer for the prospective and retrospective cohorts of patients are listed in Supplementary Tables 1 and 2, respectively. Staging was based on the TNM system by the American Joint Committee on Cancer. Fourteen healthy donors from the Health Sciences Authority of Singapore were included as controls.

### Blood and tissue processing

Blood was collected by venepuncture into an EDTA vacutainer. Peripheral blood mononuclear cells (PBMCs) were isolated and cryopreserved, as previously described [22]. Briefly, PBMCs were separated from erythrocytes on a Ficoll-Paque PLUS (GE Healthcare) gradient. Residual erythrocytes were lysed with 3 mL RBC Lysis Buffer (G-Biosciences) for 5 min at room temperature. Viable cells were counted manually using a hemocytometer based on the Trypan Blue exclusion method. Cryostocks were prepared by resuspending the PBMCs in heat-inactivated fetal bovine serum (FBS; Gibco, Thermo Fisher Scientific) supplemented with 10% v/v DMSO (Sigma-Aldrich) to a concentration of 5 × 10^6^ cells/mL and gradually frozen to − 80 °C in Mr. Frosty devices. Tissue dissociation medium was prepared by supplementing RPMI 1640 medium (Gibco) with 1 X GlutaMAX (Gibco), 0.2 mg/mL Type IV collagenase (Gibco) and 0.5 mg/mL DNAse I (Sigma-Aldrich). The tumor sample was finely cut and incubated in dissociation medium at 37 °C for 20 min. Then, the dissociated cells were sequentially passed through 70 and 30 µm cell strainers. Erythrocytes were lysed using RBC Lysis Buffer, and viable cells were counted and cryopreserved as described above.

### FCM

Complete medium was prepared by supplementing RPMI 1640 medium with 1 X GlutaMAX, 55 µM β-mercaptoethanol (Gibco), and 10% v/v heat-inactivated FBS. Cryostocks were rapidly thawed in a 37 °C water bath, and the DMSO was slowly diluted with the drop-wise addition of pre-warmed complete T cell medium. The cells were then washed twice, and resuspended in fresh medium. Supplementary Table 3 lists the antibodies and other reagents required for FCM. The PBMCs were first labeled with Fixable Viability Dye eFluor^™^ 455UV (eBioscience, Thermo Fisher Scientific) for live/dead discrimination. Then, Fc receptors were blocked with Human TruStain FcX^™^ (BioLegend) before additional surface staining was performed. Matched isotype control antibodies were included, where required. Single color compensation controls were prepared using Ultracomp eBeads (eBioscience). The samples were read in an LSRFortessa^™^ (BD Biosciences) flow cytometer. Data analysis was performed using FlowJo^®^ V10 software.

Single Tregs were imaged using the DEPArray^™^ NxT platform (Menarini Silicon Biosystems). The Tregs were initially isolated in bulk from PBMCs using a CD4^+^CD25^+^CD127^dim/−^ Regulatory T Cell Isolation Kit II, human (Miltenyi Biotec). The Tregs were surface stained and then fixed with 4% formaldehyde (Invitrogen, Thermo Fisher Scientific). Then, the Tregs were washed twice with SB115 buffer (Menarini Silicon Biosystems) before loading into the DEPArray^™^ NxT cartridge (Menarini Silicon Biosystems) for acquisition. The antibodies used for DEPArray^™^ NxT sorting are listed in Supplementary Table 4.

### Enzyme-linked immunosorbent assay (ELISA)

Soluble CD30 (sCD30) levels in serum samples of CRC patients (*n* = 23) and healthy subjects (*n* = 23) were measured using the commercially available Human CD30 ELISA Kit (ab236711, Abcam) according to the manufacturer’s instructions.

### Tissue Microarray (TMA) and multiplex immunohistochemistry/immunofluorescence (mIHC/IF)

Tumor regions for tissue microarray (TMA) construction were selected based on pathological assessment, with > 50% of the sample representing tumor area. For each sample, two or three representative tumor cores of 1 mm diameter were transferred from donor FFPE tissue blocks to recipient TMA blocks using an MTA-1 Manual Tissue Arrayer (Beecher Instruments, Inc., Sun Prairie, WI, USA).

mIHC/IF was performed using an Opal Multiplex fIHC kit (Akoya Bioscience, Menlo Park, California, USA), as previously described [23–35]. Slides were labeled with DAPI, Foxp3, OX40, and CD30, followed by appropriate secondary antibodies (see Supplementary Table 5 for details). The detailed protocol has been described previously [33].

Briefly, FFPE tissue sections were cut onto Bond Plus slides (Leica Biosystems, Richmond, Illinois, USA) and heated at 60 °C for 20 min [36]. Tissue slides were then subjected to deparaffinization, rehydration, and heat-induced epitope retrieval (HIER) using a Leica Bond Max autostainer (Leica Biosystems, Melbourne, Australia), before endogenous peroxidase blocking (Leica Biosystems, Newcastle, UK). Slides were incubated with primary antibodies followed by application of polymeric HRP-conjugated secondary antibodies (Leica Biosystems Newcastle, UK). An appropriate Opal fluorophore-conjugated TSA (Akoya Bioscience, Menlo Park, California, USA) was then added at 1:100 dilution. Slides were rinsed with washing buffer after each step. Following TSA deposition, slides were again subjected to HIER to strip the tissue-bound primary/secondary antibody complexes and ready for labeling of the next marker. These steps were repeated until all markers were labeled. Finally, spectral DAPI (Akoya Bioscience, Menlo Park, California, USA) was added at 1:10 dilution. Slides were mounted in ProLong Diamond Anti-fade Mountant (Molecular Probes, Life Technologies, USA) and cured in the dark at room temperature for 24 h. Images were acquired for each case (viable tumor regions were selected by pathologists) using a Vectra 3 pathology imaging system microscope (Akoya Bioscience, Menlo Park, California, USA) and then analyzed and scored by a pathologist with inForm software (version 2.4.2; Akoya Bioscience, Menlo Park, California, USA) [24, 37, 38] and HALO TM (Indica Labs) [39, 40].

### Statistics

Two-tailed paired *t* test, two-tailed unpaired *t* test with Welch’s correction, or one-way ANOVA with Tukey’s multiple comparison was performed on data collected from FCM analysis using GraphPad Prism V7.03 (GraphPad Software, Inc), where appropriate. Patient survival data were obtained from medical records. Disease-free survival (DFS) was defined as the time from surgery to recurrence; overall survival (OS) was defined as the duration the patient remained alive after surgery. Statistical analysis of mIHC data was performed using SPSS for Windows, Version 18. Survival outcomes were estimated with the Kaplan–Meier analysis and compared between groups by Cox regression adjusted for age at diagnosis, and the grade and stage of cancer. *: *P* ≤ 0.05; **: *P* ≤ 0.01; ***: *P* ≤ 0.001; ****: *P* ≤ 0.0001; ns: *P* > 0.05.

## Results

### Tumor-infiltrating Tregs express specific cytokine receptors and co-signaling molecules at varying levels

**Fig. 1:**
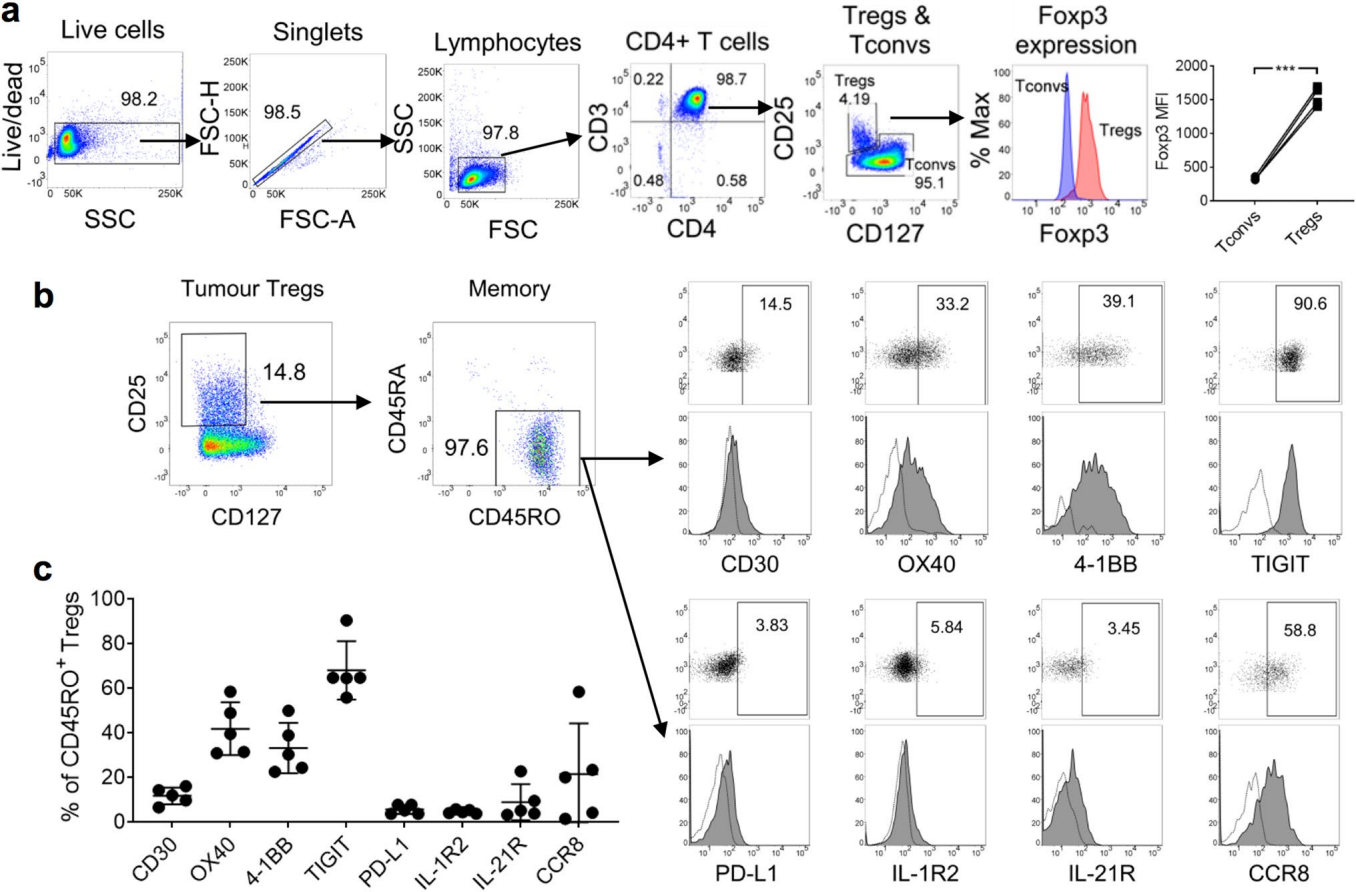
Tumor-infiltrating Tregs in CRC were predominantly CD45RO^+^ and expressed co-stimulatory molecules and cytokine receptors at varying levels. **a** Gating strategy for the analysis of Tregs by FCM. Data from four healthy subjects are presented as an example. CD4^+^ T cells were negatively selected from PBMCs using magnetic beads. Tregs were defined as CD4^+^CD25^+^CD127^–^ cells; Foxp3 expression was significantly higher than Tconvs. MFI: geometric mean fluorescence intensity. Statistical analysis was performed using the two-tailed paired *t* test. *** denotes *P* < 0.001. **b** Phenotype of tumor-infiltrating Tregs. Tumor specimens from five CRC patients were dissociated into single-cell suspensions for analysis. Representative plots are shown. Numbers within plots represent percentages. Gates for dot plots were drawn based on isotype control antibodies. Dotted histogram: isotype control antibody; filled histogram: antigen-specific antibody. **c** Proportions of CD45RO^+^ tumor-infiltrating Tregs expressing individual protein markers

De Simone et al. [21] reported that specific cytokine receptors and co-signaling molecules are differentially expressed by tumor-infiltrating Tregs in CRC patients recruited in Italy., Based on these findings, we set out to ascertain whether CRC patients from our South-East Asian cohort also expressed these markers. We first set up our Treg gating strategy by multicolor FCM using PBMCs from four healthy subjects. We defined Tregs as CD4^+^CD25^+^CD127^−^ cells, based on a previous report [41]. Compared with conventional CD4^+^ T cells (Tconvs), these Tregs exhibited markedly higher Foxp3 marker expression (Fig. 1a), thereby confirming they were bona fide Tregs. Using this same Treg gating strategy, we next examined the surface expression of eight protein markers (CD30, OX40, 4-1BB, TIGIT, PD-L1, IL-1R2, IL-21R, and CCR8) on tumor-infiltrating Tregs of five CRC patients from our South-East Asian cohort. Strikingly, 96.7 ± 1.86% of Tregs expressed the CD45RO memory marker (Fig. 1b), suggesting that nearly all of these Tregs were antigen-experienced. By gating on these memory Tregs, we observed a low frequency of PD-L1^+^, IL-1R2^+^, or IL-21R^+^ cells (5.74 ± 2.04%, 4.76 ± 0.85%, and 9.01 ± 8.10%, respectively), a moderate frequency of CCR8^+^ or CD30^+^ Tregs (21.61 ± 22.74 and 11.85 ± 3.76%, respectively), and a relatively high frequency of OX40^+^, 4-1BB^+^, or TIGIT^+^ Tregs (41.95 ± 11.86%, 33.31 ± 11.31%, and 68.15 ± 13.08%, respectively; Fig. 1c). In summary, we detected previously identified cytokine receptors and co-signaling molecules on tumor-infiltrating Tregs in our South-East Asian patients with CRC. These surface signature markers were expressed at varying levels.

### Circulating CD45RO^+^ Tregs from patients with CRC express CD30 and OX40

Having established the presence of signature markers on the Tregs from our tumor samples, we questioned whether these markers were differentially expressed by circulating Tregs in CRC patients (*n* = 25) and healthy subjects (n = 14). Gating on CD45RA^−^CD45RO^+^ circulating Tregs (as per our earlier strategy), we determined that PD-L1, IL-1R2, IL-21R, and CCR8 were generally absent (Supplementary Fig. 1a) and that the proportions of expressing cells were similar between CRC patients and healthy subjects (Supplementary Fig. 1b–e). The expression of 4-1BB was low, and the expression of TIGIT was high; however, their expression did not differ significantly between CRC patients and healthy subjects (Supplementary Fig. 1f–g).

**Fig. 2:**
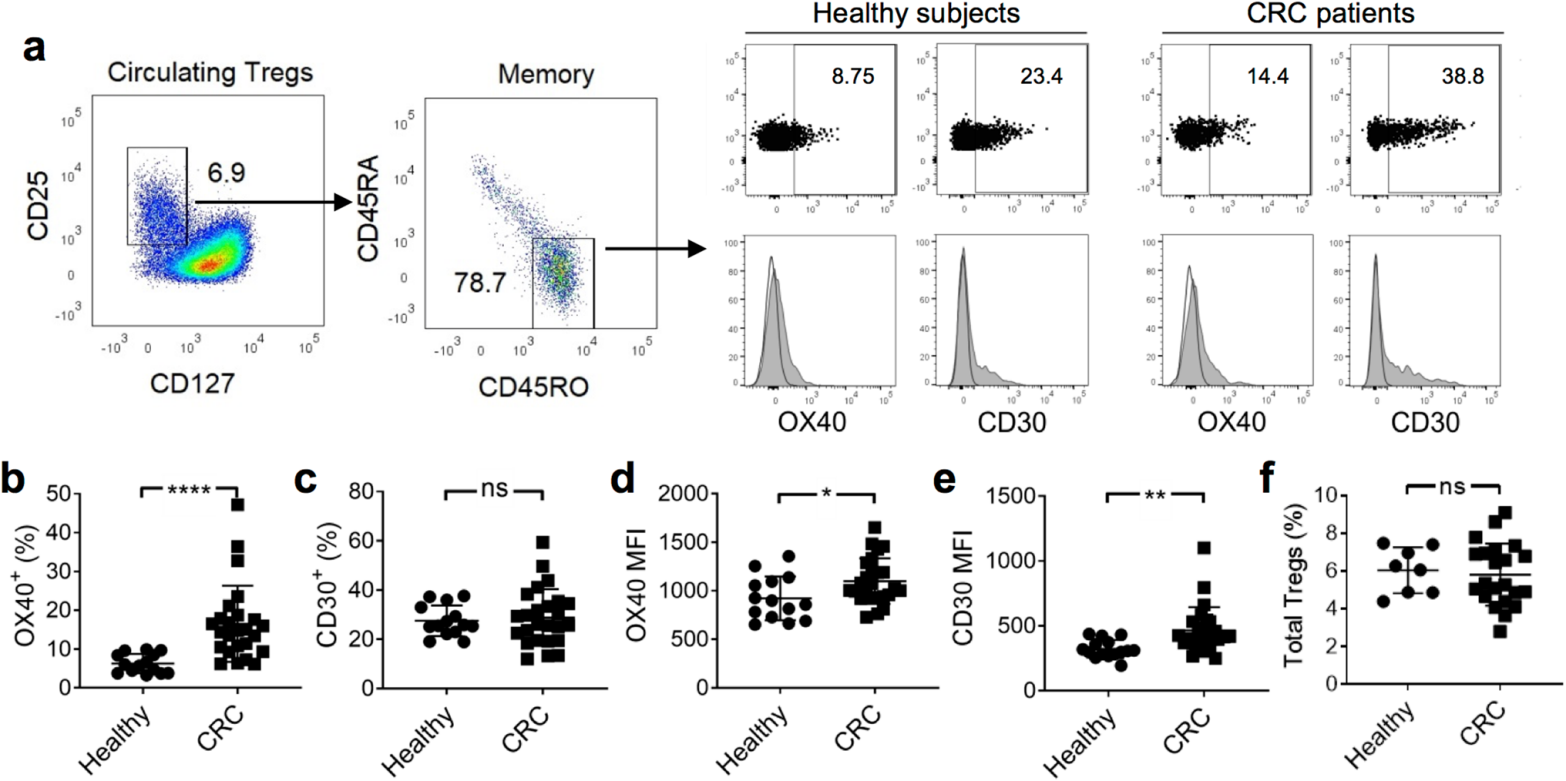
Increased expression of CD30 and OX40 by circulating CD45RO^+^ Tregs from CRC patients. **a** Representative dot plots and histograms of CD30 and OX40 expression on CD45RA^−^CD45RO^+^ Tregs from healthy subjects (*n* = 14) and CRC patients (*n* = 25) are shown. Numbers within plots represent percentages. Gates for dot plots were drawn based on isotype control antibodies. White-filled histogram: isotype control antibody; gray-filled histogram: antigen-specific antibody. **b**, **d** Proportions of circulating OX40^+^ Tregs and corresponding MFI in healthy subjects and CRC patients. **c**, **e** Proportions of circulating CD30^+^ Tregs and corresponding MFI in healthy subjects and CRC patients. **f** Proportions of circulating CD4^+^CD25^+^CD127^−^ Tregs in healthy subjects and CRC patients. Means ± SD are shown. Statistical analyses in **b**–**f** were performed using the two-tailed unpaired *t* test with Welch’s correction. *, **, and **** denote *P* < 0.05, *P* < 0.01, and *P* < 0.0001, respectively

Additionally, we detected the expression of both CD30 and OX40 in all subjects (Fig. 2a). Notably, the proportion of OX40-expressing Tregs was significantly higher in CRC patients than in healthy subjects (16.52% ± 1.96 vs. 6.28% ± 2.48, respectively; Fig. 2b). Similarly, the OX40 MFI was significantly higher in CRC patients than in healthy subjects (Fig. 2d). While the proportion of CD30-expressing Tregs was similar between CRC patients and healthy subjects, the CD30 MFI was significantly higher in CRC patients than in healthy subjects (Fig. 2c, e, respectively). No significant difference was observed in serum sCD30 levels between CRC patients (*n* = 23) and healthy subjects (*n* = 23, Supplementary Fig. 2), suggesting no preferential cleavage of CD30 in CRC patients following cellular activation. Interestingly, the levels of total circulating Tregs (expressed as a percentage of CD4^+^ T cells) did not differ between the two groups (Fig. 2f), indicating that the increase in the OX40^+^ subset in CRC patients was not due to elevated Treg levels in general. Cumulatively, these studies establish that circulating CD45RO^+^ Tregs from CRC patients and healthy subjects may express markers found in tumor-infiltrating Tregs, including TIGIT, OX40, and CD30. Given that the expression of CD30 and OX40 were significantly higher in CRC patients compared to healthy subjects; these markers might have a potential application in a blood-based method for detecting CRC.

### A CD45RO^+^CD30^+^OX40^+^ Treg subset is elevated in CRC

**Fig. 3:**
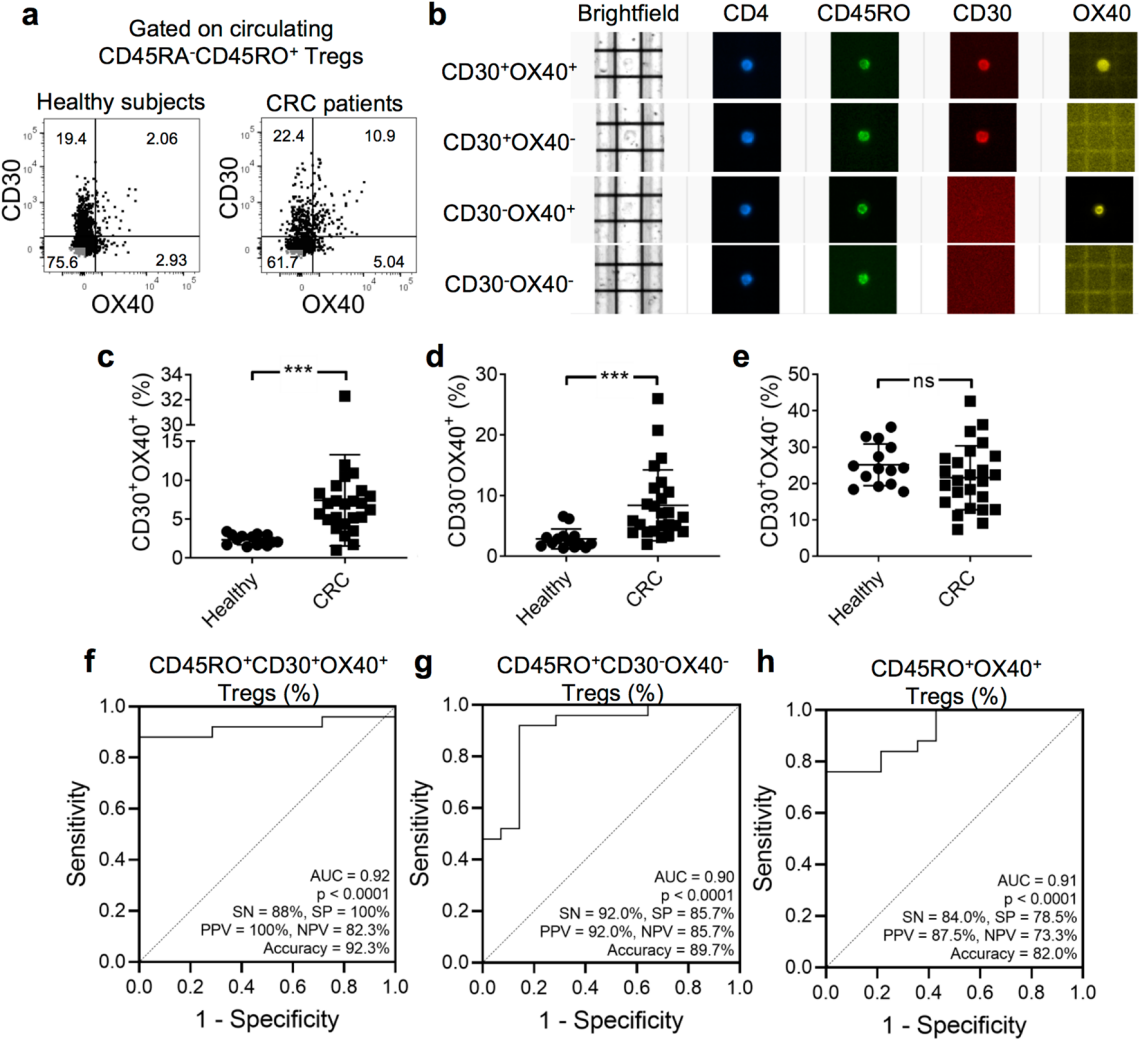
CRC is associated with an increase in circulating CD45RO^+^CD30^+^OX40^+^ Tregs. **a** Representative dot plots showing co-expression of CD30 and OX40 by CD45RO^+^ Tregs. Gates were drawn based on isotype control antibodies. **b** Treg cells imaged by the DEPArray™ NxT digital sorting system. Each row represents a single cell imaged in brightfield and four fluorescent channels. **c**–**e** Comparison of frequencies of circulating CD30^+^OX40^+^, CD30^–^OX40^+^, or CD30^+^OX40^−^CD45RO^+^ Tregs between healthy subjects (*n* = 14) and CRC patients (*n* = 25). Means ± SD are shown. Statistical analysis was performed using the two-tailed unpaired *t* test with Welch’s correction. *** denotes *P* < 0.001. **f** ROC analysis of % circulating CD45RO^+^CD30^+^OX40^+^ Tregs in discriminating healthy subjects and CRC patients, with AUC = 0.92, sensitivity = 88%, specificity = 100%, positive predictive value = 100%, negative predictive value = 82.35%, accuracy = 92.3% at a trade-off value of 3.44%. **g** ROC analysis of % circulating CD45RO^+^CD30^−^OX40^+^ Tregs in discriminating healthy subjects and CRC patients, with AUC = 0.90, sensitivity = 85.7%, specificity = 92%, positive predictive value = 92%, negative predictive value = 85.7%, accuracy = 89.7% at a trade-off value of 3.37%. **h** ROC analysis of % circulating CD45RO^+^OX40^+^ Tregs in discriminating healthy subjects and CRC patients, with AUC = 0.91, sensitivity = 84%, specificity = 78%, positive predictive value = 87.5%, negative predictive value = 73.3%, accuracy = 82% at a trade-off value of 8.87%

Based on the expression of CD30 and OX40, we were able to identify three subpopulations of Tregs by FCM: CD30^+^OX40^+^, CD30^+^OX40^−^, and CD30^−^OX40^+^ Tregs (Fig. 3a), of which the CD30^+^OX40^+^ subset was the rarest. Using the DEPArray^™^ NxT system, which was designed for imaging and sorting of single cells, we further confirmed the presence of CD30^+^ and/or OX40^+^ Tregs (Fig. 3b). The proportion of CD30^+^OX40^+^ and CD30^−^OX40^+^ expressing cells was significantly elevated in CRC patients compared with healthy subjects (CD30^+^OX40^+^, 7.41% ± 1.17 vs. 2.34% ± 0.17; CD30^−^OX40^+^, 8.41% ± 1.16 vs. 2.87% ± 0.43; Fig. 3c, d). A similar percentage of CD30^+^OX40^−^ Tregs was observed between CRC patients and healthy subjects (Fig. 3e). To the best of our knowledge, this finding represents the first time that CD45RO^+^CD30^+^OX40^+^ Tregs have been identified as a distinct subpopulation with an association with CRC.

We next asked whether Treg frequency may be used to discriminate between patients with CRC and healthy subjects. ROC analyses of all Treg subsets revealed that CD45RO^+^CD30^+^OX40^+^, CD45RO^+^CD30^−^OX40^+^, and CD45RO^+^OX40^+^ Tregs could clearly discriminate between CRC patients and healthy subjects with an AUC > 0.9 and *P* < 0.0001 (Fig. 3f–h). Among these Treg subsets, CD45RO^+^CD30^+^OX40^+^ Tregs achieved the highest accuracy of 92.3% with an AUC of 0.92, a sensitivity of 88%, a specificity of 100%, a positive predictive value of 100%, a negative predictive value of 82.35%, and a trade-off value of 3.44%. The remaining Treg subsets were poorly discriminative (AUC < 0.8; Supplementary Fig. 3a, b). Altogether, our data suggest a potential role for circulating CD45RO^+^CD30^+^OX40^+^ Tregs in CRC diagnosis.

### A high density of tumor-infiltrating CD30^+^OX40^+^ Tregs predicts improved OS

A high density of tumor-infiltrating Foxp3^+^ Tregs frequently correlates with improved OS following surgical resection for CRC [12–15]; thus, Foxp3^+^ Tregs are regarded as an important prognostic indicator. In light of our findings on the strong association between CD30^+^OX40^+^ Tregs and CRC, we interrogated subpopulations of tumor-infiltrating Foxp3^+^ Tregs to identify which ones, if any, correlate with patient survival.

**Fig. 4:**
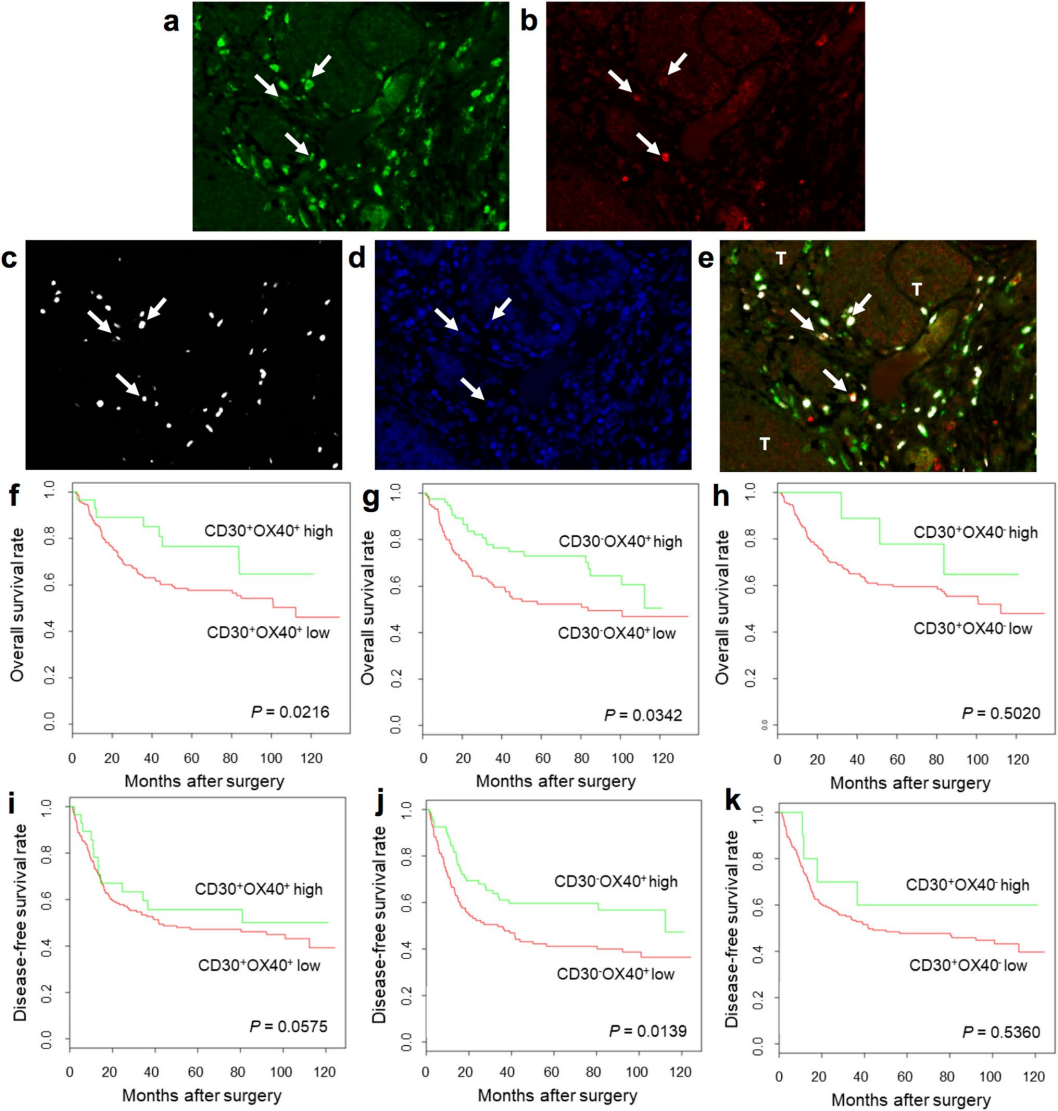
**A** high density of tumor-infiltrating CD30^+^OX40^+^ Tregs is associated with improved OS after surgery**. a**–**e** Multiplex-IHC/IF of representative tumor section at 200X magnification. Color scheme is as follows: CD30 (green), OX40 (red), Foxp3 (white), and DAPI (blue). Single staining for CD30 (**a**), OX40 (**b**), Foxp3 (**c**), DAPI (**d**), and triple-staining for CD30, OX40, and Foxp3 (**e**) are shown. White arrow: CD30^+^OX40^+^ Treg cell; T: tumor island. **f**–**k** Kaplan–Meier curves of OS and DFS of CRC patients who underwent surgery. Patients were segregated into high (green curve) and low (red curve) groups based on the density of different tumor-infiltrating Tregs: **f**, **i** CD30^+^OX40^+^ Tregs, **g**, **j** CD30^–^OX40^+^ Tregs, **h**, **k** CD30^+^OX40^−^ Tregs. Survival outcomes were compared between groups using univariate analysis. *P* value is indicated in each graph

We examined the archival tumor tissues from 217 patients who underwent surgery and were subsequently monitored for a minimum of 5 years. We simultaneously stained the tumor sections with Foxp3, CD30, and OX40 (Fig. 4a–e), and then determined the numbers of CD30^+^OX40^−^, CD30^−^OX40^+^, and CD30^+^OX40^+^ Tregs. Interestingly, analysis of CD30^+^OX40^+^Foxp3^+^ Tregs revealed significant differences in OS, with a high Treg density being associated with improved survival (Fig. 4f; *P* = 0.0216). We found no significant difference in disease-free survival (DFS; Fig. 4i; *P* = 0.0575). Nevertheless, multivariate analysis using Cox regression further confirmed the finding and revealed statistically significant differences in OS, but not DFS, between patients with a high or low density of CD30^+^OX40^+^Foxp3^+^ Tregs (Tables 1 and 2; OS, HR: 0.43, *P* = 0.0428; DFS, HR: 0.75, *P* = 0.3810).

**Table 1:** Multivariate analysis of the effect of novel Tregs subsets on OS, adjusted for tumor grade, stage, and patients’ age

Factor	OR (95% CI)	*P* value
**CD30**^**+**^**OX40**^**+**^** Tregs**		
Low	Reference	
High	0.43 (0.19, 0.97)	0.0428*
**CD30**^**−**^**OX40**^**+**^** Tregs**		
Low	Reference	
High	1.05 (0.64, 1.71)	0.8498
**CD30**^**+**^**OX40**^**−**^** Tregs**		
Low	Reference	
High	0.95 (0.44, 2.01)	0.8843

^*^P < 0.05 indicates a statistically significant difference

**Table 2:** Multivariate analysis of the effect of novel Tregs subsets on DFS, adjusted for tumor grade, stage, and patients’ age

Factor	OR (95% CI)	*P* value
**CD30**^**+**^**OX40**^**+**^** Tregs**		
Low	Reference	
High	0.75 (0.40, 1.42)	0.3810
**CD30**^**−**^**OX40**^**+**^** Tregs**		
Low	Reference	
High	0.73 (0.45, 1.18)	0.1990
**CD30**^**+**^**OX40**^**−**^** Tregs**		
Low	Reference	
High	1.21 (0.43, 3.42)	0.7140

^*^P < 0.05 indicates a statistically significant difference

Kaplan–Meier curves of OS and DFS rates revealed significant differences between patients with a high or low density of CD30^−^OX40^+^Foxp3^+^ Tregs (Fig. 4g, j; *P* = 0.0342 and *P* = 0.0139, respectively); however, multivariate analysis using Cox regression failed to establish statistically significant differences in survival outcomes (Tables 1 and 2; OS, *P* = 0.8498; DFS, *P* = 0.1990). Similarly, for the comparison of patients with a high or low density of CD30^+^OX40^−^Foxp3^+^ Tregs, Kaplan–Meier and Cox regression analyses did not demonstrate significant differences in survival rates (Fig. 4h, k, Tables 1 and 2). Altogether, these data support a role for tumor-infiltrating CD30^+^OX40^+^, but not CD30^−^OX40^+^ or CD30^+^OX40^−^, Foxp3^+^ Tregs in predicting OS of CRC patients.

## Discussion

Several studies have shown that patients with CRC exhibit elevated levels of Tregs in tumors and peripheral blood [42–45]. Notably, CRC tumor-infiltrating Tregs can be distinguished by the increased expression of a panel of signature markers, including CD30, IL1R2, IL21R, OX40, CCR8, PD-L1, TIGIT, and 4-1BB [21]. In view of these results, we asked whether circulating Tregs from CRC patients might also exhibit such a distinct expression profile and possibly serve as a cancer biomarker.

We started our investigation with tumor-infiltrating Tregs and observed a preponderance of CD45RO^+^ cells, suggesting that nearly all Tregs had encountered cognate antigen and had been activated. Indeed, CRC tumors are reportedly immunogenic [46], and Tregs are among the most expanded T cell population within tumors [47]. Among the signature markers we examined, we consistently observed marked expression of the co-signaling molecules CD30, OX40, 4-1BB, and TIGIT, while PD-L1, IL-1R2, IL-21R, and CCR8 were either marginally or inconsistently expressed. The presence of CD30, OX40, and 4-1BB, and the upregulation of TIGIT were in line with the memory phenotype of tumor-infiltrating Tregs, given the association of these molecules with activated T cells [48–52].

In light of these observations from tumor-infiltrating Tregs, we focused on circulating CD45RO^+^ Tregs. Here, we found that tumor signature markers were poorly expressed by these circulating Tregs, suggesting that the environmental triggers present in CRC tumors and the blood were largely distinct. Nevertheless, we did observe a significant increase in CD30 and OX40 activation markers expression in circulating Tregs in CRC patients, suggesting an increase in Treg activity. CD30 and OX40 are quickly downregulated after T cell activation [49, 51, 53], suggesting a recent encounter with cognate antigen, possibly of tumor origin. We further demonstrated that the frequency of CD30^+^OX40^+^ and CD30^−^OX40^+^ Tregs was higher in the peripheral blood of CRC patients than healthy subjects. Importantly, ROC analyses of the various Treg subsets identified in this study revealed that CD30^+^OX40^+^ Tregs provide the strongest discrimination between CRC patients and healthy subjects. Taken together, our results indicate the potential application of circulating CD45RO^+^CD30^+^OX40^+^ Tregs in a blood-based method for CRC diagnosis.

Our observation that the total number of circulating Tregs did not differ between CRC patients and healthy subjects contradicts earlier findings [42–44]. One possible reason for this difference could be the lack of a consistent Treg definition across different studies, which prevents a direct comparison of the findings. Although Foxp3 is essential to maintain the Treg lineage, it is by no means unique because activated human CD4^+^ Tconvs also transiently express Foxp3 [54]. Given the various definitions of Tregs (CD4^+^CD25^+^, CD4^+^CD25^hi^, or CD4^+^Foxp3^+^ cells), our study used a more stringent definition of Treg, i.e., CD4^+^CD25^+^CD127^−^ [41], which we further demonstrated to uniformly and highly express Foxp3. We also cannot rule out patient population differences as a cause of discrepancy: patients recruited in earlier studies were of Caucasian or Japanese origin, whereas we recruited patients of South-East Asian origin [42–44].

With regard to tumor-infiltrating Tregs, their prognostic significance in the context of CRC is well established; a high infiltration of Foxp3^+^ Tregs is frequently associated with improved OS after surgical resection [12–15, 55], although their association with DFS is less clear [56–58]. These findings seem counter-intuitive, given that Tregs have strong immunosuppressive activity and are generally considered to inhibit anti-tumor immune responses [59]. Further work is needed to fully understand this paradigm. The apparent benefit of infiltrating Tregs in CRC might stem from direct and indirect effects. For example, Tregs might be beneficial as a result of the suppression of an unresolved inflammatory response that drives tumor progression [12]. Indeed, CRC is mostly linked to environmental causes rather than heritable genetic changes, and one major risk factor is chronic intestinal inflammation [60]. Alternatively, Treg density can positively correlate with the prevalence of other cell types, such as CD8^+^ T cells, which exert direct tumor killing [14, 56].

Here, we interrogated tumor-infiltrating Treg subsets to identify associations with patient survival after surgery. Multivariate analyses indicated that neither CD30^+^OX40^−^ nor CD30^−^OX40^+^ Tregs were linked to any survival outcome, whereas a high density of CD30^+^OX40^+^ Tregs was associated with improved OS but not DFS. The latter finding supports previous studies showing an association between Foxp3^+^ Tregs and OS but not DFS [12–15, 55–58]. These findings are interesting for a couple of reasons: firstly, the CD30^+^OX40^+^ subset might be an important player in shaping the association between Foxp3^+^ Tregs and improved OS, although we cannot rule out other undefined subsets at this stage. Secondly, they suggest functional heterogeneity among Treg subsets with a differential impact on patient survival. Indeed, there is increasing appreciation that Tregs are not a uniform population but are comprised of phenotypically and functionally diverse subsets [16, 17, 20]. It has also been argued that Foxp3 is not a true signature for Tregs as conventional T cells also express Foxp3 upon activation, albeit transiently and at a significantly lower level [18, 61]. We speculate that the combination of CD30, OX40, and Foxp3 markers might improve the definition for Tregs with actual suppressive function, and may permit a more precise determination of the effect of Tregs on patient survival.

However, the exact functional relevance of OX40 and CD30 co-expression on Foxp3-intact human Tregs cells is not fully understood. Both CD30 and OX40 are co-signaling molecules of the tumor necrosis factor receptor superfamily (TNFRSF). The absence of OX40 and CD30 co-stimulatory signals prevents CD4 T cell-driven autoimmune disease [62] and anti-tumor CD8 T cell responses [63] can be achieved in Foxp3-deficient mice. There are also studies showing that exposure to anti-OX40 promotes Treg cell response, while others suggest that anti-OX40 mAbs block the suppressive functions of Tregs [64]. Whether CD30 and OX40 are required to function together or differentially to regulate Treg responses and anti-tumor immunity warrants further study.

To our knowledge, this is the first study to provide evidence of a CD30^+^OX40^+^ Treg subset and its association with CRC and clinical outcome. CRC-infiltrating CD30^+^OX40^+^ Tregs exhibited a prognostic value, whereas their CD30^+^OX40^–^ and CD30^−^OX40^+^ counterparts did not, and could serve as a biomarker for predicting patient survival. Although CRC-infiltrating CD30^+^OX40^+^ Treg subset could be a robust indicator of patient survival, their clinical utility may be challenging to implement. Tissue biopsy is required to evaluate the frequency of CRC-infiltrating CD30^+^OX40^+^ Tregs. The invasive nature of tissue biopsy is an obstacle to frequent sampling, and re-biopsy is impractical for most patients with high-risk profiles. Ideally, routine clinical assessment should be performed in a minimally invasive manner through blood-circulating biomarkers.

Here, we showed that circulating CD30^+^OX40^+^ Tregs were present in our CRC patients of South-East Asian origin, at a significantly higher level than healthy subjects and could strongly distinguish CRC patients from healthy subjects, suggesting a diagnostic role. However, it is challenging to study blood-circulating CD30^+^OX40^+^ Tregs at single-cell resolution due to their rarity (7.41% of total CD45RO^+^ Tregs). Most of the mainstream single-cell technologies (e.g., 10X Genomics, Drop-Seq, FCM) require samples with high cellularity for phenotyping and molecular characterization. In contrast, the imaging-based digital cell sorting DEPArray™ NxT technology can distinguish, select, and sort rare target circulating CD30^+^OX40^+^ Tregs with high resolution and purity. The unique CD30^+^OX40^+^ Treg subset that can be validated at single-cell resolution, in both Caucasian and Asian cohorts, in tissue and liquid biopsies, may be used as a potential immune-oncology biomarker to diagnose and prognosticate CRC. Further studies are warranted to validate these findings in a larger cohort, and other gastrointestinal tract cancers such as gastric and esophageal cancers.

## Supplementary Information

Supplementary file1 (PDF 575 kb)

## Data Availability

All data generated or analyzed during this study are included in this published article and its supplementary information files.
