# Supplementary material for: CD30+OX40+ Treg is associated with improved overall survival in colorectal cancer
Source: Cancer Immunol Immunother. 2021 Feb 2;70(8):2353–65. doi: 10.1007/s00262-021-02859-x (PMC8289785; doi:10.1007/s00262-021-02859-x)
Supplement: Supplementary file 1 — Supplementary file1 (PDF 575 kb) [file 262_2021_2859_MOESM1_ESM.pdf]

Supplemental Material

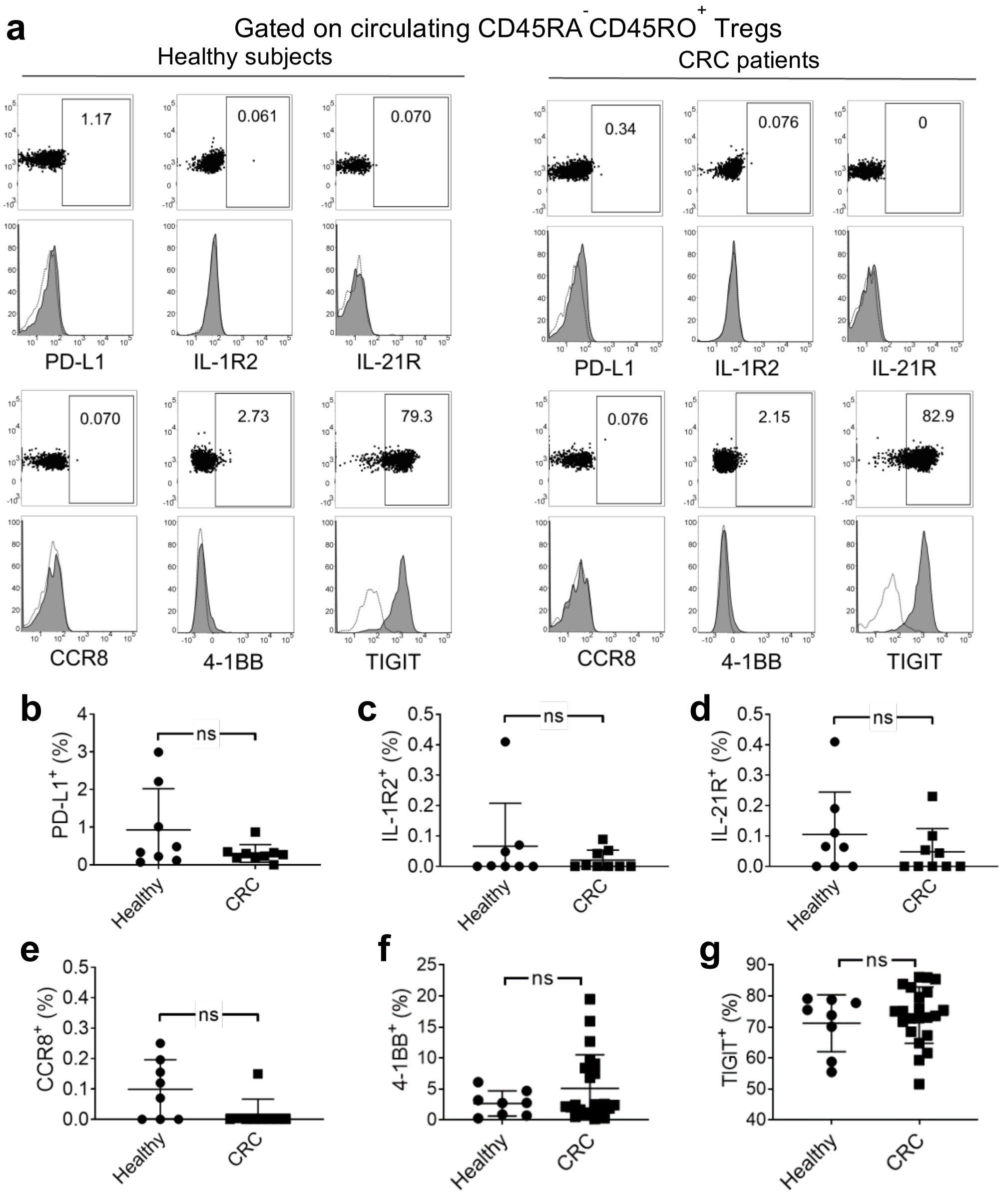

Supplementary Fig. 1 Frequencies of circulating CD45RO+ Tregs expressing PD-L1, IL-1R2, IL-21R, CCR8, 4-1BB, or TIGIT in healthy subjects and CRC patients. **a** Representative dot plots and histograms of healthy subjects (n = 14) and CRC patients (n = 25). Numbers within plots represent percentages. Gates were drawn based on isotype control antibodies. Dotted histogram: isotype control antibody; filled histogram: antigen-specific antibody. **b-g** Comparison of frequencies of CD45RO+ Treg subsets expressing the indicated markers between healthy subjects and CRC patients. Means ± SD are shown. Statistical analysis was performed using the two-tailed unpaired *t*-test with Welch's correction. ns indicates not significant.

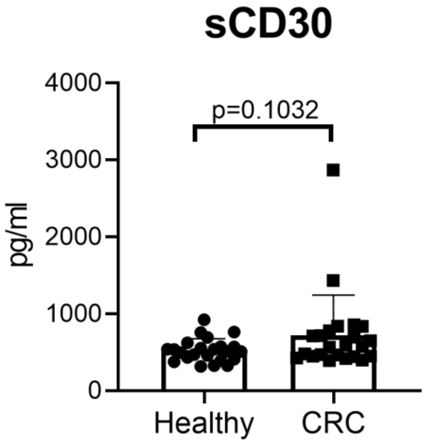

Supplementary Fig. 2 Soluble CD30 (sCD30) levels in the serum of healthy subjects and CRC patients. sCD30 levels were measured in the serum of healthy subjects (n = 23) and CRC patients (n = 23) using commercially available ELISA kit according to the manufacturer's instructions. Means ± SD are shown. Statistical analysis was performed using the two-tailed unpaired *t*-test with Welch's correction.

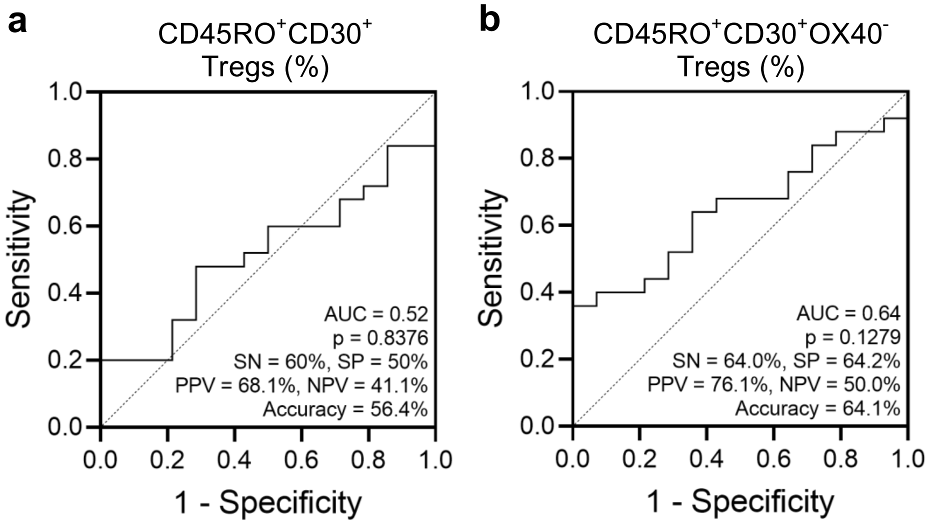

Supplementary Fig. 3 ROC analyses of circulating $CD45RO^+$ Treg subsets in discriminating CRC patients (n = 25) and healthy subjects (n =14). **a** Frequency of total $CD30^+$ Tregs, with AUC = 0.52, sensitivity = 60%, specificity = 50%, positive predictive value = 68.1%, negative predictive value = 41.1%, accuracy = 56.4% and a trade-off value of 25.57%. **b** Frequency of total $CD30^+OX40^-$ Tregs, with AUC = 0.64, sensitivity = 64%, specificity = 64.2%, positive predictive value = 76.1%, negative predictive value = 50%, accuracy = 64.1% and a trade-off value of 23.49%.

Supplementary Table 1 Demographics, tumor histology, and cancer stage of 30 CRC patients for FCM studies.

Patient #	Gender	Age	Site of primary tumor	Histology	pTNM: T	pTNM: N	pTNM: M	Stage	Prior treatment	Sample analyzed
1	M	81	Rectum	Adenocarcinoma NOS	3	2a	0	IIIB	No	Tumor
2	F	60	Rectum	Moderately differentiated adenocarcinoma	3	1a	0	IIIB	No	Tumor
3	M	65	Sigmoid colon	Adenocarcinoma NOS	4b	1c	0	IIIC	Tx bladder cancer 5 yr. earlier	Tumor
4	M	47	Sigmoid colon	Mucinous adenocarcinoma	4a	0	0	IIB	Tx colon cancer 7 yr. earlier	Tumor
5	F	57	Sigmoid colon	Adenocarcinoma NOS	3	0	0	IIA	No	Tumor
6	F	67	Rectosigmoid Colon	Adenocarcinoma, NOS	4a	2	0	IIIC	Yes	Blood
7	M	76	Splenic Flexure	Adenocarcinoma, NOS	3	0	1	IV	Yes	Blood
8	F	71	Sigmoid Colon	Adenocarcinoma	NA	NA	1	IV	Yes	Blood
9	M	52	Rectum	Adenocarcinoma, NOS	3	0	1	IV	Yes	Blood
10	F	56	Caecum	Medullary carcinoma	NA	NA	1	IV	Yes	Blood
11	M	62	Sigmoid	Adenocarcinoma	4	2a	1	IV	Yes	Blood
12	F	42	Rectum	Adenocarcinoma, NOS	4a	2	1	IV	Yes	Blood
13	F	66	Ascending colon	Adenocarcinoma	2	2a	1	IV	Yes	Blood
14	F	48	Sigmoid	Adenocarcinoma	3	2b	1	IV	Yes	Blood
15	M	46	Rectosigmoid	Adenocarcinoma	4a	2b	1	IVA	Yes	Blood
16	F	69	Caecum	Medullary carcinoma	4b	2a	NA	IV	Yes	Blood
17	M	56	Rectum	Adenocarcinoma	3	1	1	IV	Yes	Blood
18	M	78	Rectosigmoid	Adenocarcinoma	4a	1c	0	IIIB	Yes	Blood
19	F	32	Rectosigmoid	Adenocarcinoma	NA			NA	Yes	Blood
20	F	51	Sigmoid	Adenocarcinoma	NA			NA	Yes	Blood
21	M	63	Sigmoid	Adenocarcinoma	3	0	0	IIA	Yes	Blood
22	F	57	Transverse colon	Adenocarcinoma	4	2	0	IIIB	Yes	Blood
23	M	72	Rectosigmoid	Adenocarcinoma	NA			III	Yes	Blood
24	F	68	Mid rectal	Adenocarcinoma	3	1b	1	IV	No	Blood
25	M	49	Anorectal	Adenocarcinoma	3	2a	0	IVA	No	Blood
26	M	50	Descending colon	Adenocarcinoma	4	2	1	IV	Yes	Blood

27	F	67	Splenic flexure	Adenocarcinoma	3	2	1	IVA	Yes	Blood
28	F	40	Anal	Adenocarcinoma	NA			IV	Yes	Blood
29	M	71	Rectosigmoid	Adenocarcinoma	NA			NA	Yes	Blood
30	M	79	Sigmoid Colon	Adenocarcinoma	4	0	0	IIB	Yes	Blood

NA: information not available, NOS: Not Otherwise Specified

Supplementary Table 2 Demographics, tumor histology, and cancer stage of 217 CRC patients for tissue microarray and multiplex-IHC/IF studies.

Factors	Patient number
**Gender**	
Female	98
Male	119
**Race**	
Chinese	169
Indian	9
Malay	15
Other	24
**Tumor grade (T)**	
1/2	15
3	140
4/4a/4b	57
N.D.	5
**Lymph node involvement (N)**	
N0	96
N1	47
N2	72
N.D.	2
**Distant metastasis (M)**	
M0	162
M1	45
N.D.	10
**Histologic grade**	
Moderate	168
Poor	19
Well	9
N.D.	21
**Location of tumor**	
Anorectal	1
Ascending colon	18
Caecum	9
Descending colon	14
Hepatic flexure	6
Rectosigmoid	22
Rectum	66
Sigmoid	48
Splenic flexure	3
Transverse colon	20
N.D.	10
**Lymphovascular invasion (LVI)**	
Absent	118
Present	90
N.D.	9

N.D.: not determined, from the pathology reports, which are mainly from the earlier years.

Supplementary Table 3 Reagents for FCM.

#	Reagent	Antibody clone; origin	Manufacturer	Catalogue number	Stock concentration (µg/ml)	Working concentration (µg/ml)
1	Fixable Viability Dye eFluor™ 455UV	Not applicable	eBioscience	65-0868-14	Not available	1:1000 dilution
2	Human TruStain FcX™	Not applicable	BioLegend	422302	Not available	1:10 dilution
3	BV510-αCD3	OKT3; mouse	BioLegend	317332	30	1.5
4	AF700-αCD4	OKT4; mouse	BioLegend	317426	500	10
5	PerCP-eFluor 710-αCD25	4E3; mouse	eBioscience	46-0257-42	12	0.6
6	PE-Cy7-αCD127	RDR5; mouse	eBioscience	25-1278-42	100	5
7	Super Bright 702-αCD45RA	HI100; mouse	eBioscience	67-0458-42	12	0.6
8	BV650-αCD45RO	UCHL1; mouse	BioLegend	304232	100	5
9	PE-αCD30	Ber-H2; mouse	eBioscience	12-0309-42	12	1.25
	PE-IgG1, isotype control	P3.6.2.8.1; mouse	eBioscience	12-4714-81	200	1.25[a]
10	APC-αOX40	Ber-ACT35; mouse	BioLegend	350008	200	15
	APC-IgG1, isotype control	P3.6.2.8.1; mouse	eBioscience	17-4714-81	200	15[a]
11	BV421-α4-1BB	4B4; mouse	BioLegend	309820	100	5
	BV421-IgG1, isotype control	MOPC-21; mouse	BioLegend	400158	100	5[a]
12	APC-αTIGIT	MBSA43; mouse	eBioscience	17-9500-42	25	1.25
	APC-IgG1, isotype control	P3.6.2.8.1; mouse	eBioscience	17-4714-81	200	1.25[a]
13	BV785-αPD-L1	29E.2A3; mouse	BioLegend	329736	120	6
	BV785-IgG2b, isotype control	MPC-11; mouse	BioLegend	400356	100	6[a]
14	BV421-αIL-21R	2G1-K12; mouse	BioLegend	347809	100	5
	BV421-IgG1, isotype control	MOPC-21; mouse	BioLegend	400158	100	5[a]
15	FITC-αIL1-R2	34141; mouse	Invitrogen	MA5-23662	Not available	1:20 dilution
	FITC-IgG1, isotype control	Clone not available; mouse	Invitrogen	GM4992	Not available	1:20 dilution[a]
16	APC-αCCR8	191704; rat	R&D systems	FAB1429A-100	10	0.5
	APC-IgG2b, isotype control	141945; rat	R&D systems	IC013A	10	0.5[a]

[a] To match the working concentration of respective antigen-specific antibody.

Supplementary Table 4 Antibodies for DEPArray™ Nxt sorting.

#	Antibody	Clone; origin	Manufacturer	Catalogue number	Stock concentration (µg/ml)	Working concentration (µg/ml)
1	BV421-αCD4	OKT4; mouse	BioLegend	317434	70	3.5
2	BB515-αCD45RO	UCHL1; mouse	BD	564529	Not indicated	1:20 dilution
3	PE-αCD30	Ber-H2; mouse	eBioscience	12−0309−42	12	0.6
4	APC-αOX40	Ber-ACT35; mouse	BioLegend	350008	200	10

Supplementary Table 5 Reagents for m-IHC/IF.

#	Reagent	Antibody clone; origin	Manufacturer	Catalogue number	Cellular location	Stock concentration (µg/ml)	Working concentration (µg/ml)
1	DAPI dye	Not applicable	Perkin Elmer	FP1490	Nucleus	Not available	1:500 dilution
2	αFoxp3	236A/E7; mouse	Abcam	ab20034	Nucleus	1000	5
3	αOX40	Ber-ACT35; mouse	eBioscience	14-1347-82	Surface membrane	500	5
4	αCD30	Ber-H2; mouse	Dako	M0751	Surface membrane	Not available	1:50 dilution
